# Genetic Markers of Genome Rearrangements in *Helicobacter pylori*

**DOI:** 10.3390/microorganisms9030621

**Published:** 2021-03-17

**Authors:** Mehwish Noureen, Takeshi Kawashima, Masanori Arita

**Affiliations:** 1 Department of Genetics, SOKENDAI University, Yata 1111, Mishima 411-8540, Shizuoka, Japan; mnoureen@nig.ac.jp; 2 Bioinformation and DDBJ Center, National Institute of Genetics, Yata 1111, Mishima 411-8540, Shizuoka, Japan; takeshik@nig.ac.jp; 3 RIKEN Center for Sustainable Resource Science, 1-7-22 Suehiro, Tsurumi, Yokohama 230-0045, Kanagawa, Japan

**Keywords:** *Helicobacter pylori*, inversion breakpoints, repeats, insertion sequences

## Abstract

*Helicobacter pylori* exhibits a diverse genomic structure with high mutation and recombination rates. Various genetic elements function as drivers of this genomic diversity including genome rearrangements. Identifying the association of these elements with rearrangements can pave the way to understand its genome evolution. We analyzed the order of orthologous genes among 72 publicly available complete genomes to identify large genome rearrangements, and rearrangement breakpoints were compared with the positions of insertion sequences, genomic islands, and restriction modification genes. Comparison of the shared inversions revealed the conserved genomic elements across strains from different geographical locations. Some were region-specific and others were global, indicating that highly shared rearrangements and their markers were more ancestral than strain—or region—specific ones. The locations of genomic islands were an important factor for the occurrence of the rearrangements. Comparative genomics helps to evaluate the conservation of various elements contributing to the diversity across genomes.

## 1. Introduction

*Helicobacter pylori* (*H. pylori*) has infected nearly half of the world’s population and may cause a wide range of diseases from mild gastritis to gastric cancer [1,2]. Since 1994, the bacterium has been categorized as a type I carcinogen by the World Health Organization, and since then, many strains have been genome-sequenced around the world [3]. Its infection usually starts during a person’s childhood and remains for their lifetime [4]. The mode of inheritance is still unclear, but *H. pylori* is considered to have co-evolved with *Homo sapiens* since its original migration “out of Africa” [5,6]. The rate of mutation and recombination of *H. pylori* is one of the fastest among bacteria, possibly to enable its flexible host adaptation [7,8]. Due to its rapid evolution, its genomic signatures can be used as anonymous markers to characterize human history.

Compared to frequent genetic variations (mutations, insertions, or deletions), genome rearrangements (inversions and translocations) are rarer markers for delineating co-evolution. Genomic rearrangements keep the genetic repertoire intact without gene gain or loss [9] and theoretically do not alter *H. pylori’s* survival fitness within the host. Flanking genes may be inserted or deleted in association with (or after) rearrangements, but the evidence of large rearrangements is harder to erase from the genome than any other small-scale genetic variations.

For example, the highest incidences of gastric cancer are observed in East Asia, and *H. pylori* in Asia forms a different clade from European and American ones [5,6]. The absence of Asia-specific gene losses or mutations suggests that a larger-scale maker is more associated with pathogenicity such as cancer and gastritis.

Molecular mechanisms causing rearrangements have been explained with several genetic factors such as repeat and insertion sequences [10,11]. Repeat sequences are the cause of genetic recombination, and the average repeat size is 53 and 100 base pairs (bp) for *Methanococcus jannaschii* and *H. pylori*, respectively [12]. There are direct repeats (DRs) and inverted repeats (IRs), and the former is considered more common [13]. In bacteria, repeat-deficient genomes seem stable, and more repeats lead to more rearrangements [13,14].

Insertion sequences (ISs), also called IS elements, are short transposable DNA fragments. ISs have been found extensively in bacterial genomes [15], often around large inversions [16,17]. In *H. pylori*, total five ISs, from IS605 to IS609, have been documented in detail [18,19,20,21]. The IS605 was the first to be reported, as the element splitting the *H. pylori’s* virulence region (cag pathogenicity island) in the rearranged strain NCTC11638 [22]. It was found in one third of *H. pylori* strains and contains two open reading frames (ORFs), orfA and orfB [18]. IS606 is similar to IS605, and the amino acid identity of two ORFs with those of IS605 is approximately 25% [18]. Similarly, IS607 and IS608 carry two ORFs, but they contain the overlap for 27 bp and 30 bp, respectively [19,20]. Finally, IS609 carries four ORFs (orf1, orf2, orfA, orfB). The gene products of the orfA in the five ISs are grouped into two subfamilies, whether encoding serine recombinases (IS607, IS609) or not (IS605, IS606, IS608) [21]. For the orfB gene, IS606, IS606, IS607, and IS608 form a large group of unknown function and only IS609 is separate.

We have previously reported 41 non-trivial genome inversions in 72 publicly available strains. Among the inversions, 18 were strain-specific and 23 were shared. The shared inversions were numbered from R1 to R23 throughout this work. Among these inversions, R1–R16 were shared in different geographical locations, and R17–R23 were region-specific. For example, the reference strain 26695 and eight related strains (26695-1CL, 26695-1CH, 26695-1, 26695-1MET, 26695, Rif1, Rif2, and 26695-1) contained five inversions (R1–R5), two of which (R1 and R2) were nested [23]. Seven strains (P12, Shi417, Shi169, Puno135, Cuz20, Lithuania75, and Aklavik117) were devoid of shared inversions. In the current report, we provide a detailed analysis on the relationship between molecular markers with these rearrangements and discuss their chronological ordering and the possible relation to the *H. pylori* pathogenicity.

## 2. Materials and Methods

### 2.1. Sequence Materials and Identification of Rearrangements

A total of 72 *H. pylori* complete genomes were obtained from the GenBank/ENA/DDBJ repository: (1) East Asia annotated as NY40, F30, ML3, ML1, UM299, UM298, UM032, UM037, UM066, F32, oki128, XZ274, OK310, 52, F16, oki673, oki154, oki828, oki898, oki112, oki102, oki422, F57, 26695-1CH, 26695-1CL, 26695-1, Hp238, OK113; (2) South America annotated as Sat464, Shi112, Shi169, Shi417, Cuz20, PeCan18, PeCan4, Puno120, Puno135, SJM180, v225d; (3) North America annotated as 7C, 29CaP, Aklavik117, Aklavik86, 26695–1, 26695-1MET, J166, J99, ELS37; (4) Europe annotated as B38, B8, HUP-B14, Rif1, Rif2, 26695, P12, 26695, G27, Lithuania75, 2017, 2018, 908; (5) Africa annotated as SouthAfrica20, SouthAfrica7, Gambia94/24; (6) India annotated as India7, Santal49; (7) Australia annotated as BM013A, BM013B, BM012A, BM012B, BM012S; and 8) others of unknown location annotated as 83 and 35A. We did not use Aklavik86 because it contained too many rearrangements.

Orthologous genes were identified by Protein BLAST (version 2.2.29+, *e*-value < 1 × 10^−5^) using the bidirectional best-hits criterion. Gene clusters of sizes less than 70 genomes were discarded. For each genome, the gene (cluster) ordering was first recorded. Next, the ordering in 72 genomes were used to generate the consensus ordering using the majority rule: for each gene cluster, the most likely upstream and downstream gene clusters were determined. According to this consensus ordering, all gene clusters were newly assigned a serial number corresponding to a generic ordering [23]. When these new numbers were mapped to each genome, rearrangement breakpoints became evident as gaps in the gene numbers. Gaps of more than two were taken into consideration (i.e., single and double gene losses were ignored), and the corresponding inversions were located using the breakpoint analysis.

Number gaps in the gene ordering were often shared among genomes. The estimation algorithm for rearrangement with manual curation was detailed in our previous report [23]. In short, single genome-specific inversions were first identified and fixed. Then, shared inversions were progressively identified and fixed from the least shared ones. For complex rearrangements, neighboring genes were manually investigated to identify the time course of genome evolution. Identified rearrangements were also detailed in our previous report [23].

GenBank accession numbers for insertion sequences (IS605, IS606, IS607, IS608, and IS609) are U60177, U95957, AF189015, AF357224, and AY639112, respectively. Identification of these sequences was performed using Blastn (Match/Mismatch scores of 1, −2 with linear gap cost; Word size 28).

### 2.2. Identification of Sequence Repeats

Direct and inverted repeats were identified using the Unipro UGENE software version 1.29.0 [24]. Parameters for the Find repeats utility were as follows: window size: 25 bp, minimum identity per window 100%, minimum distance between repeats 0 bp, and maximum distance between repeats 1,000,000 bp. The relative location of repeat sequences and the rearrangements were investigated manually.

### 2.3. Genomic Islands

IsalndViewer4 webserver was used to obtain the information regarding the presence of genomic islands (GIs) in *H. pylori* strains [25]. This webserver had the precomputed results for several genomes. GI information of all the *H. pylori* strains in this study were obtained from the precomputed results. The relative location of genomic islands and the rearrangements were investigated manually.

## 3. Results and Discussion

### 3.1. Genome Rearrangements 

**Figure 1:**
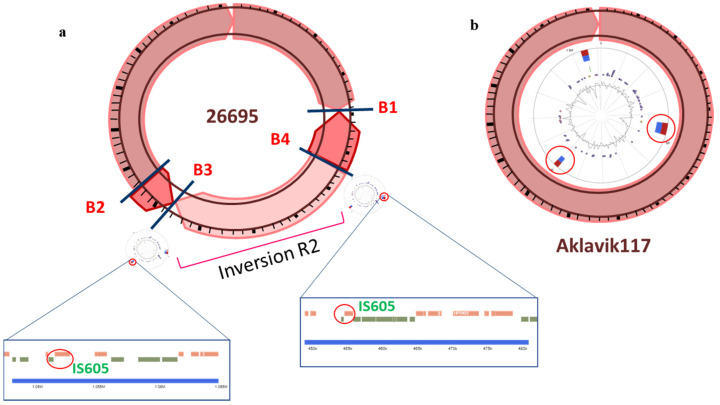
(**a**) Graphical representation of the *Helicobacter pylori* 26695 strain. Four breakpoints are indicated by crossing lines and the corresponding labels represent the breakpoint number (B1–B4). Two genomic islands (GIs) were identified in this strain that are present at the location of two breakpoints B3 and B4. Within these GIs, IS605 was present as an inverted repeat. (**b**) Graphical representation of *H. pylori* Aklavik117 strain. This strain possessed the two GIs almost at the same location as in (**a**), but it lacked the insertion sequence (IS) elements in these GIs and the inversion R2 was absent.

Some inversions occurred more frequently compared to others. The inversions R3, R5, R6, R12, and R13 were present in more than 10 strains from different geographical locations. The genomic regions around these inversions can be called rearrangement hotspots. For example, the reference strain 26,695 possessed two nested inversions (R1 and R2) in comparison with Aklavik117, a strain from North America. The inner inversion R2 was associated with GIs with inverted IS605 repeat as its possible cause (Figure 1). Two African strains (SouthAfrica20 and SouthAfrica7) without the R2 inversion also lacked IS605 in their GIs. The conserved existence of the GIs indicated their early formation, followed by the uptake of the ISs and the R2 inversion event.

However, not all the insertion sequences were associated with GIs (Supplementary Figure S1). The type and the number of insertion sequences varied among strains, and 24 strains were devoid of intact IS elements. In strains with many IS elements, around half of them were associated with GIs, but the number of GIs also did not correlate with the number of IS elements. In order to discuss the relationship in more detail, we introduce the notion of breakpoints.

### 3.2. Inversion Breakpoints

**Figure 2:**
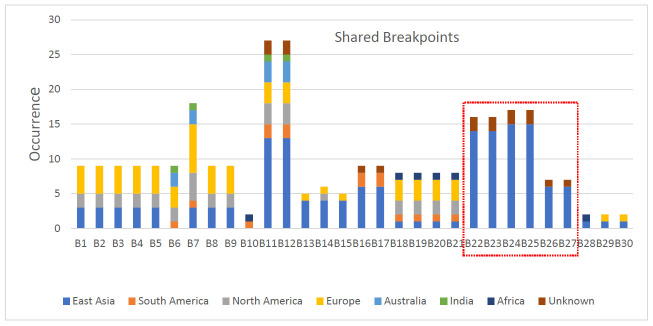
Distribution of shared breakpoints among strains from different geographical locations. Breakpoints are designated as B1–B30. B22–B27 can be regarded as East-Asia-specific.

Two terminals of an inversion are referred to as breakpoints. Seventy-one breakpoints, designated as B1–B71, were identified in the analyzed strains, corresponding to the 41 inversions. The number of breakpoints did not match the doubled number of inversions because of their reuse: 13 breakpoints were involved in more than one inversion. Among the 71 breakpoints, B1–B30 were shared among the strains from different geographical locations whereas B31–B44 were shared among the strains from the same geographical location (region-specific) and B45–B71 were strain-specific. Among the shared breakpoints (B1–B30), B22–B27 were observed in large number of East Asian strains along with a few strains with unknown geographical location; we called them East-Asia-specific breakpoints. Similarly, some breakpoints were observed only in strains from particular geographical locations. Figure 2 illustrates the distribution of shared breakpoints among strains from different geographical locations. The largest number of breakpoints was 10 in strains from East Asia and Australia [23]. Detailed information about the inversion and their corresponding breakpoints is shown in Supplementary Table S1.

### 3.3. Repeat Sequences and Their Associated Inversions

In most prokaryotes, a repeat sequence of length >25 is assumed to involve in homologous recombination with statistical significance [11,26,27]. We investigated all direct and inverted repeats of length >25 nucleotides with 100% sequence identity in all strains (Supplementary File S3: Figure S2). Among the 41 inversions, 20 inversions were associated with repeats. For example, the inversion R6 was observed in 27 strains, among which 20 were associated with inverted repeats around its two breakpoints. Exceptions were four strains from Okinawa (Japan) that possessed no element at one breakpoint (B11) and a direct repeat at the other (B12) and three strains from Australia that possessed a direct repeat at one breakpoint (B11) and an inverted repeat at the other (B12).

Table 1 shows the number of associated inverted and direct repeats with inversions. The ratio of inverted versus direct repeats (IR/DR) was less than 1 (Supplementary Figure S3) and the total number (and their total length) of direct and inverted repeats was proportional to the genome size (Supplementary Figure S4) [28].

The correlation between the number of repeats and that of inversions was weak. This suggested that the occurrence of repeats was not the direct cause of inversions. Their relative position, especially the relation with GIs, seemed important for homologous recombination.

**Figure 3:**
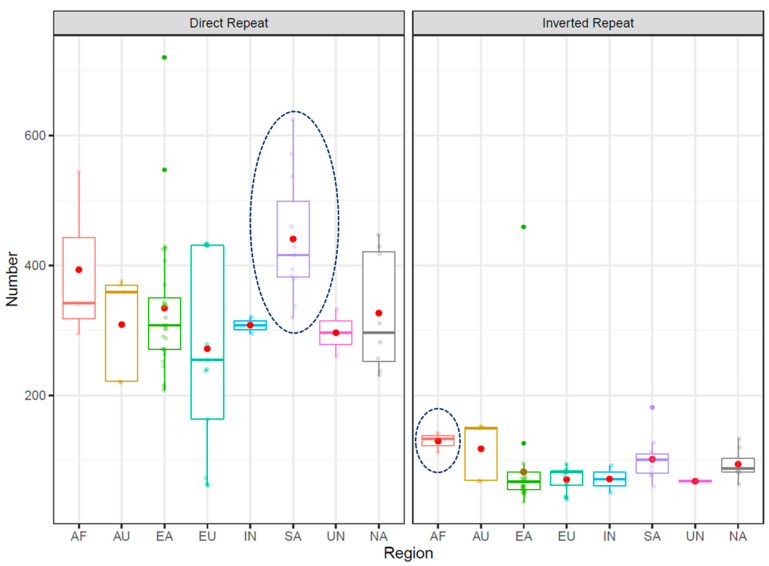
Distribution of direct and inverted repeats in different geographical regions. Region names are abbreviated (AF: Africa, AU: Australia, EA: East Asia, EU: Europe, IN: India, SA: South America, UN: region not known, and NA: North America). Red dots represent the average number of repeats identified in each region. Regions with the largest number of repeats on average are encircled.

A larger number of direct and inverted repeats were found in South American and African strains (Figure 3). The longest direct and inverted repeats of length 8,041 bp, 10,305 bp were observed in strains SouthAfrica7 (Africa) and F16 (East Asia) (Supplementary Table S2). The average size of longest repeats in each region is shown in Table 2. The least number of direct and inverted repeats was observed in the strains 2018 and F57 from Europe and East Asia, respectively. The largest number of direct and inverted repeats was found in UM037, an East Asian strain. This strain contained six inversions, among which three were associated with inverted repeats (R16, R37, and R38).

Among the different types of inversions, five world-wide, five region-specific, and seven strains-specific inversions possessed the inverted repeat around their breakpoints. Larger inversions (in terms of the number of inverted genes) possessed larger repeats. A significant positive correlation was observed between the inversion size (number of inverted genes) and the average size of repeat found around those inversions (Supplementary Figure S5).

### 3.4. Presence of Genomic Islands around Inversion Breakpoints

GIs represent regions acquired by horizontal gene transfer [27]. A varying number of genomic islands was present in the analyzed strains. Six GIs were the largest and were found in two strains: Shi112 (South America) and J99 (North America). The average number of the identified GIs was two. Most region-specific and strain-specific breakpoints were observed in the neighborhood of GIs (Table 3). In three Australian strains, four GIs were located in the neighborhood of Australia-specific breakpoints.

The most frequent global inversions, R3 and R6, were distant from any GIs but neighbored by repeat sequences. Compared to such global breakpoints, region- and strain-specific breakpoints were often neighbored with GIs. These local breakpoints seemed to have formed after the global breakpoints were established.

### 3.5. Distribution of Insertion Sequences and Their Association with Inversions

Different types of insertion sequences (IS605-IS609) have been reported in *H. pylori* [18,19,20,21]. We performed detailed analyses of these five elements around inversions (Table 4 and Supplementary File S8: Figure S6). Association between insertion sequences and breakpoints is summarized in Table 5.

Both IS605 and IS606 were found in multiple geographical regions around the widely shared breakpoints of inversion R2 (Figure 1a) and R28 respectively, with inverted repeats. IS605 was found in 16 strains and 13 of them carried two standard ORFs (orfA and orfB). Anomalies were one strain from South America (Sat464) lacking orfA and two strains (v225d from South America and 83 from Unknown) with nonsense mutations in orfB (pseudo gene). Of note, 26695 related strains possessed five copies of IS605, and the same number of IS605 were retained in distant strains of G27 (European) and UM037 (East Asia).

IS606 was present in 30 strains worldwide. It was observed in African strains. One strain (ELS37 from North America) possessed six copies, but all others possessed up to three. Eight strains in the same clade (Cuz20, Shi417, PeCan4, Shi169, Puno135, Sat464, and v225d from South America and Aklavik117 from North America) possessed orfB only; this observation indicated that the deletion of orfA occurred before the diversification of strains in America. These strains, however, possessed different numbers of IS607 and GIs. In addition, some IS606 were found within GIs whereas others were not. Therefore, the possibility of recombination between strains also remained. In two strains, Sat464 and v225d, the orfB contained a nonsense mutation.

IS607 was region-specific in South America and Australia. It was present in 15 strains, including all Australian strains. All strains except one had both orfA and orfB having an overlap of 27 bp between them [19]. In two strains, orfB contained nonsense mutation. In one East Asian strain (F16) its orfA was pseudo gene and orfB was split into two genes.

IS608 was also region-specific, mainly in South America. It was present in 13 strains, including four Peruvian strains: two from gastric cancer (PeCan4, PeCan18) and two from unknown disease state (Shi112, Cuz20) [20]. In Asia, only strains from Okinawa possessed this sequence with orfB only. In Australia, three strains possessed this sequence, but its orfB was dysfunctional.

Finally, IS609 was found in Europe and North America but not in Asia, Australia, and South America. SJM180 was classified as South America, but its phylogenetic clade showed its closeness to European strains. Complete IS609 (all four ORFs) were found in few strains only: one European and one American strains (B38 and 29CaP). Four Okinawa strains were exceptions because they possessed the complete copy of IS609 and their phylogenetic clade was closer to European strains.

Partially deleted IS elements were more likely to be outside of GIs. This indicated that IS elements were still active and transferred in/out of GIs (Supplementary Figure S1).

### 3.6. Other Molecular Elements Related to Inversions

**Figure 4:**
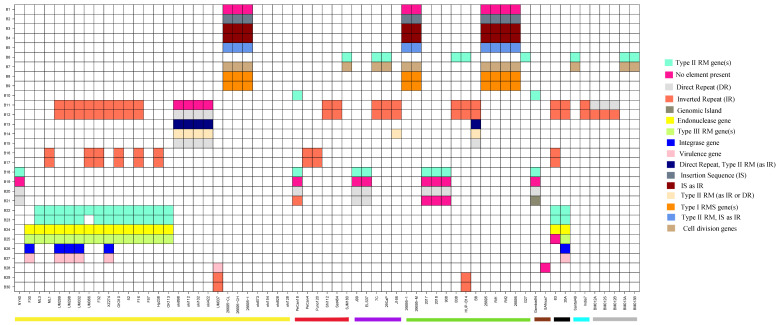
Presence of different elements around shared breakpoints at the strain level. Each column represents one strain (names indicated at the bottom) whereas each row indicates one shared breakpoint. Colored bars below strain names indicate the different regions (Yellow: East Asia, Red: South America, Purple: North America, Green: Europe, Brown: Africa, Black: Region not known, Blue: India, and Grey: Australia). Different colors in the cells represent different elements. The while cells indicate the absence of breakpoint.

In addition to the repeats, ISs, and GIs, other elements like DNA methyltransferases, restriction modification (RM) system, and virulence related genes were also searched in the neighborhood of the identified breakpoints (Figure 4). Type II RM genes were more abundant than Type I and Type III RM genes. The strains sharing the same inversion breakpoints tended to possess similar elements (see also Supplementary Table S3). Since the number of analyzed strains was small, finding the specificity of these elements with any of the disease states requires analysis on a larger scale.

## 4. Conclusions

Analysis of genome rearrangements in association with insertion sequences and repeats can reveal genome evolution in a finer scale. We have compared the strains from different geographical locations to identify the association of several genomic elements with the inversions. Most of the shared inversions possessed similar IS elements with a few exceptions. This suggests that these elements are well-conserved irrespective of the different geographical region. Restricted distributions of IS607 and IS608 indicated their relatively recent proliferation compared to IS605 and IS606, and isolation of partial IS elements from GIs indicated the important roles of GIs in distributing IS elements.

Our analysis was limited to the publicly available strains. A larger scale analysis can help us to understand the geographical distribution and association of disease with different genomic elements. Since *H. pylori* can cause different diseases, analysis of various rearrangements can lead us to identify the underlying possible causes, thus facilitating a better understanding of disease mechanisms.

## Figures and Tables

**Table 1:** Number of inverted and direct repeats associated with different types of inversions.

Inversion Type	Total Inversions	IR Associated Inversions	DR Associated Inversions
World-wide	16	5	2
Region-specific	7	4	1
Strain-specific	18	5	3

**Table 2:** Average size of longest repeats observed in each geographical region.

Region	Average Size of Longest Inverted Repeat	Average Size of Longest Direct Repeat
East Asia	2181	3425
South America	1631	4495
North America	2145	3745
Europe	2268	2756
Africa	4587	5057
India	1772	4084
Australia	2315	3033
Unknown	1357	3154

**Table 3:** Strains having genomic island(s) associated with breakpoints.

Strain	Accession	No. of GIs	GIs Associated with Breakpoints
UM037	NC_021217.3	3	1
F32	NC_017366.1	1	1
26695-1CL	NZ_AP013356.1	2	2
26695-1CH	NZ_AP013355.1	3	2
26695-1	NZ_AP013354.1	3	2
Aklavik117	NC_019560.1	4	2
26695-1	NZ_CP010435.1	3	2
26695-1MET	NZ_CP010436.1	3	2
ELS37	NC_017063.1	2	1
Rif2	NC_018938.1	3	2
Rif1	NC_018937.1	3	2
26695	NC_018939.1	3	2
26695	NC_000915.1	2	2
Gambia94/24	NC_017371.1	5	2
SouthAfrica20	NC_022130.1	4	1
India7	NC_017372.1	4	1
BM012A	NC_022886.1	5	4
BM012S	NC_022911.1	4	4
BM012B	NZ_CP007605.1	4	4

**Table 4:** Number of copies of each IS element (IS605-IS609) in all the strains. Fraction indicates an incomplete IS element. (See also Supplementary Figure S1.).

Strains	Region	IS605	IS606	IS607	IS608	IS609
NY40	East Asia	1	4	1	0	0
ML3	East Asia	0	1	0	0	0
UM032, UM298, UM299, F30, F57, ML1, UM066, OK310, 52, Hp238	East Asia	0	0	0	0	0
UM037	East Asia	5	0	0	0	0
F32	East Asia	1	0	0	0	0
XZ274	East Asia	0	0	2	0	0
F16, OK113	East Asia	0	0	1	0	0
oki128, oki154, oki673, oki828	East Asia	0	3	0	0	0
oki102, oki112, oki422, oki898	East Asia	0	0	0	0.5	1
26695-1CL, 26695-1CH, 26695-1	East Asia	5	2	0	0	0
Shi112	South America	0	0	5	2	0
Sat464	South America	0.5	0.5	1	0	0
Cuz20	South America	0	0.5	1	1	0
PeCan4	South America	0	0.5	0	1	0
PeCan18	South America	0	0	0	2	0
Puno120	South America	0	0	0	0	0
Shi169	South America	0	0.5	6	0	0
SJM180	South America	0	0	0	0	1
Puno135, Shi417	South America	0	0.5	0	0	0
v225d	South America	1	0.5	0	0	0
7C, J166	North America	0	0	0	0	0.5
29CaP	North America	0	1	0	0	4
Aklavik117	North America	0	0.5	1	0	0
26695-1, 26695-1MET	North America	5	2	0	0	0
J99	North America	0	1	0	0	0.5
ELS37	North America	0	6	1	0	0
B38	Europe	0	0	0	0	5
HUP-B14	Europe	0	0	0	1	1
Rif1, Rif2, 26,695	Europe	5	2	0	0	0
B8	Europe	0	0	0	1	0.5
G27	Europe	5	0	0	0	0
Lithuania75, P12	Europe	0	0	0	0	0
2017, 2018, 908	Europe	0	1	0	0	0.5
SouthAfrica7	Africa	0	2	0	0	0.5
Gambia94/24, SouthAfrica20	Africa	0	0	0	0	0.5
India7, Santal49	India	0	0	0	0	0
BM012A, BM012B, BM012S	Australia	0	0	9	0.5	0
BM013A, BM013B	Australia	0	0	4	0	0
83	Unknown	1	0	0	0	0
35A	unknown	0	0	0	0	0

**Table 5:** Number of insertion sequence (IS) present around different types of inversion breakpoints (BPs).

IS	World-Wide BPs	Region-Specific BPs	Strain-Specific BPs
IS605	4	0	1
IS606	3	1	0
IS607	0	3	0
IS608	0	1	0
IS609	0	0	0

## Data Availability

The complete genome sequences used in this study are available at GenBank/ENA/DDBJ (See Supplementary File 6: Table S2 for the accession numbers).

## Supplementary Materials

The following are available online at https://www.mdpi.com/2076-2607/9/3/621/s1, Figure S1: Distribution of IS elements; Table S1: Breakpoint positions in each strain; Figure S2: Occurrence of direct and inverted repeats; Figure S3: Association between direct and inverted repeats; Figure S4: Association between genome sizes and repeats; Table S2: Accession numbers of strains and repeat sizes; Figure S5: Association between gene numbers and repeats; Figure S6: Gene structure of IS elements; Table S3: Genomic elements around each breakpoint.
