# Supplementary material for: Genetic Markers of Genome Rearrangements in Helicobacter pylori
Source: Microorganisms. 2021 Mar 17;9(3):621. doi: 10.3390/microorganisms9030621 (PMC8002640; doi:10.3390/microorganisms9030621)
Supplement: Supplementary file 1 [file microorganisms-09-00621-s001.zip › Supplementary_files/Supplementary file1_Figure S1.pdf]

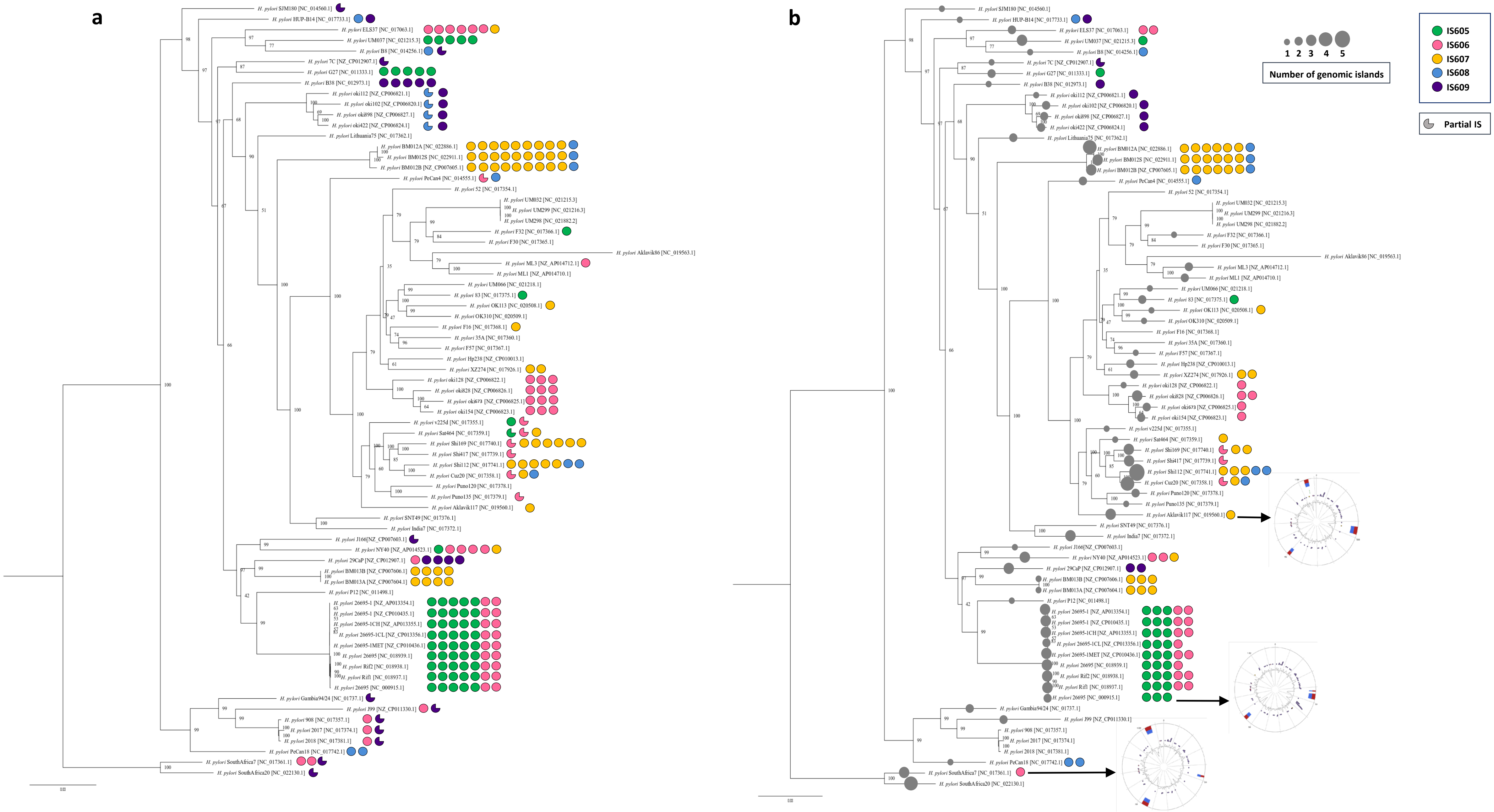

Figure S1. **(a)** Core genes phylogenetic tree along with the distribution of the five insertion sequences (IS) shown in colored circle. Different colors represent the various IS elements as shown in the top right legend; (**b)** Core genes phylogenetic tree along with the distribution of the IS elements present in the genomic islands (GIs) identified in each strain. Gray colored circles along the branches indicate the number of GIs present in a particular strain. The plot showing the location of GIs is shown for the three strains.

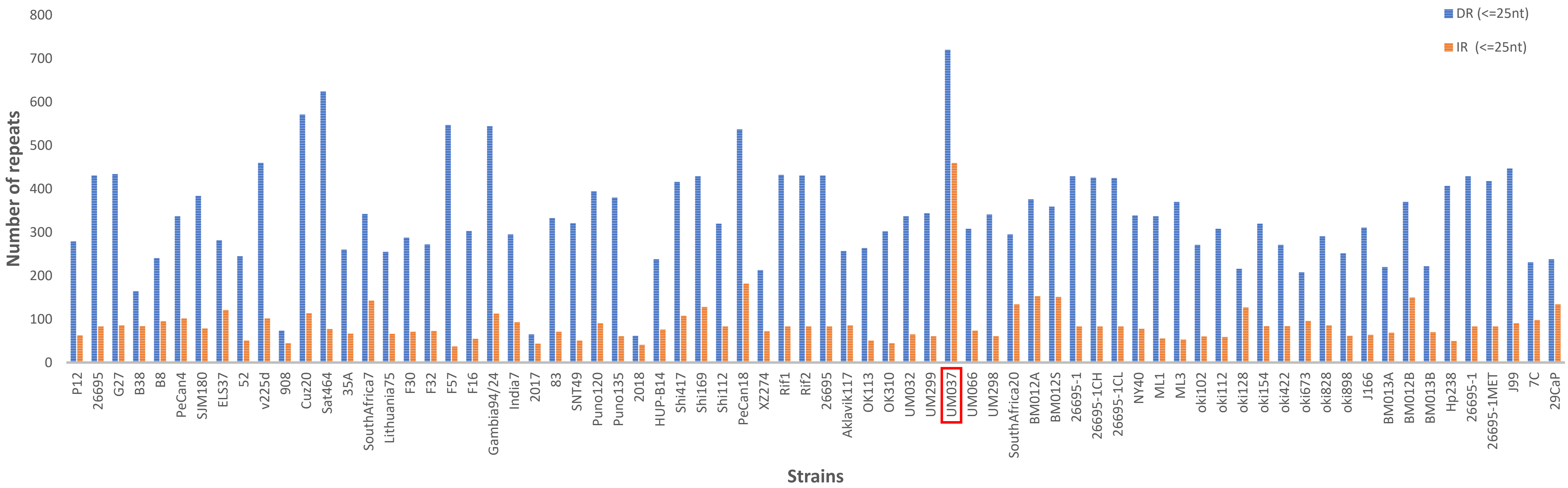

Figure S2. Occurrence of the direct and inverted repeats in each strain. Direct and inverted repeats of length >=25 nucleotide and 100 % sequence identity are shown. Strain UM037 had the highest number of direct as well as the inverted repeats among all of the analyzed strains.

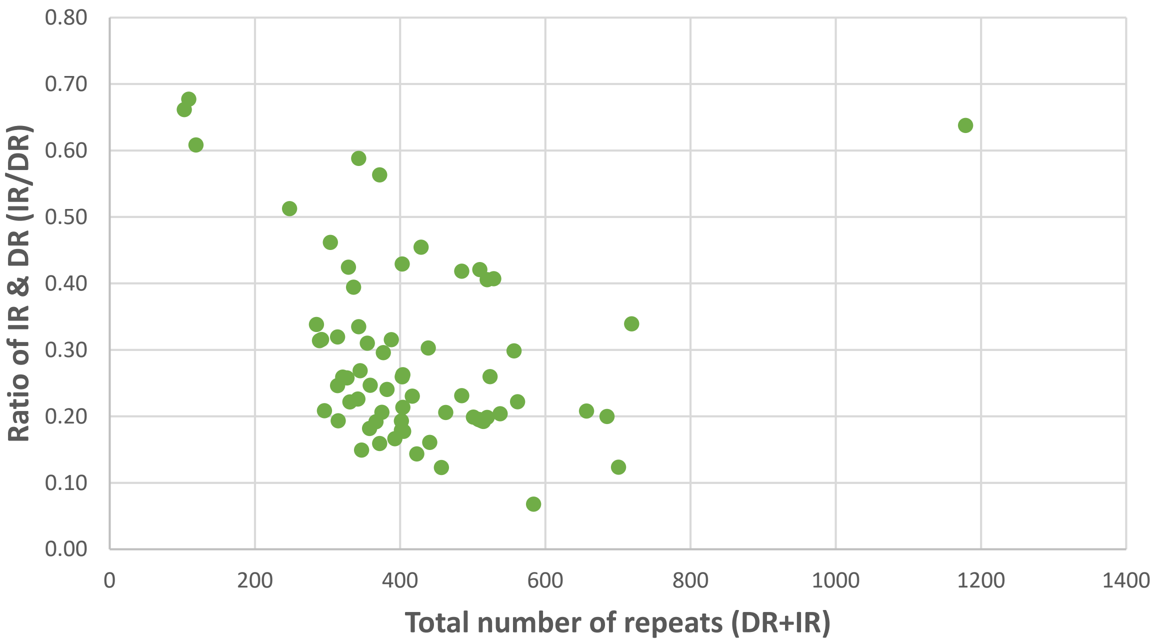

Figure S3: Distribution of the ratio of inverted repeats (IR) over direct repeats (DR). This ratio (IR/DR) less than 1 indicates the underrepresentation of inverted repeats.

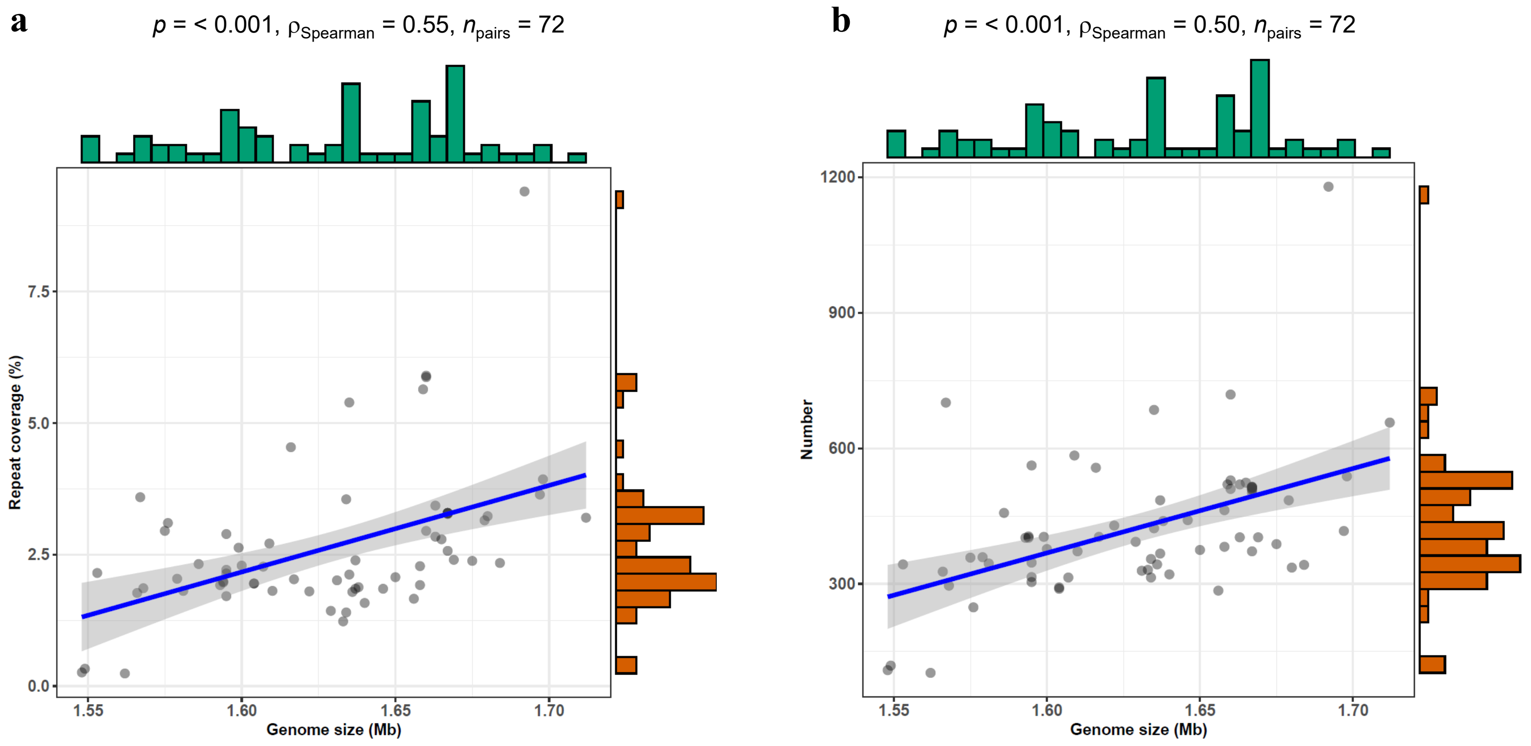

Figure S4 (a-b): a Association between genome size and repeat coverage. **b** Association between genome size and number of repeats. Positive correlation was observed for both a and b.

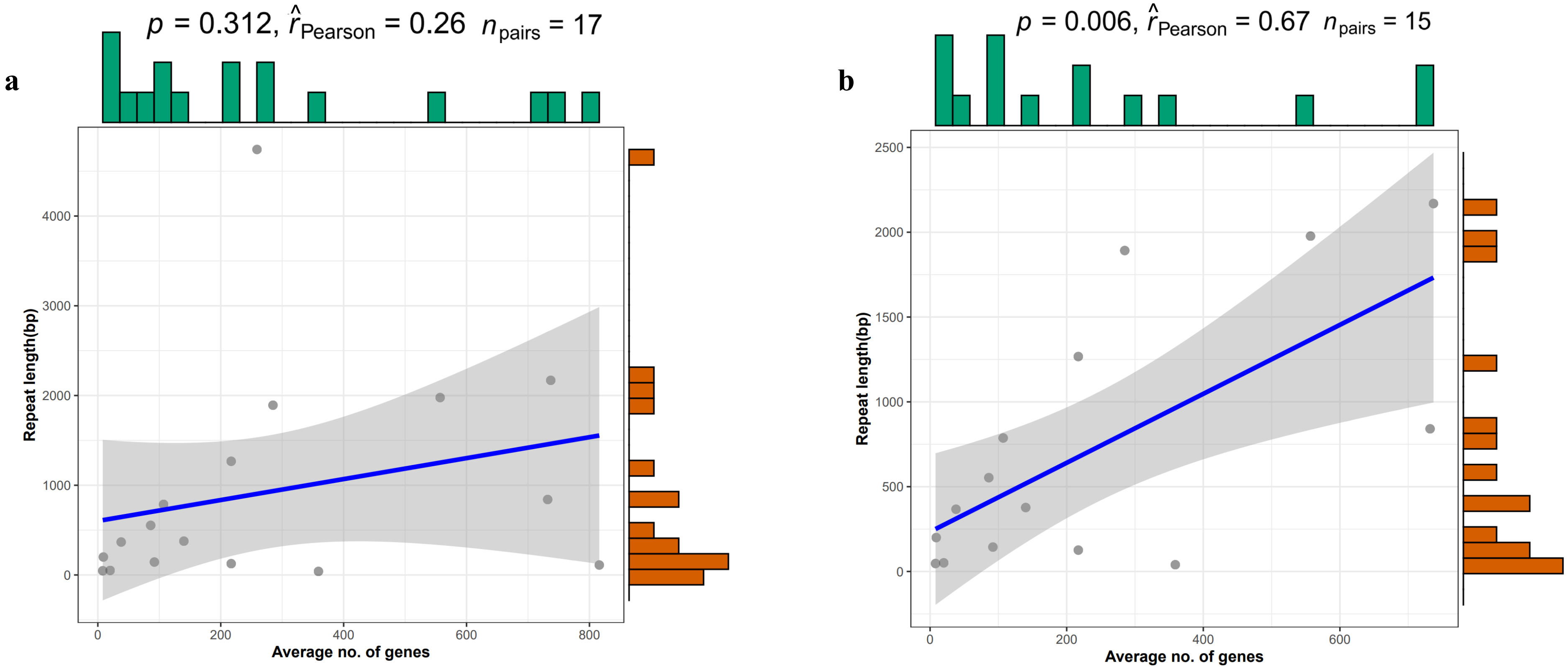

Figure S5 (a-b): a Association between average number of genes in an inversion and length of repeat present around its breakpoints. **b** Association between average number of genes in an inversion and length of repeat present around its breakpoints after removing two outliers [the inversion R7 (the inverse transposition of 22 genes when dealt as an inversion had 816 genes while the repeat was 111 bp in length) and inversion R26 (strain-specific inversion)]. A significant positive correlation was observed.
