# Supplementary material for: Genetic Markers of Genome Rearrangements in Helicobacter pylori
Source: Microorganisms. 2021 Mar 17;9(3):621. doi: 10.3390/microorganisms9030621 (PMC8002640; doi:10.3390/microorganisms9030621)
Supplement: Supplementary file 1 [file microorganisms-09-00621-s001.zip › Supplementary_files/Supplementary file3_Figure S2.pdf]

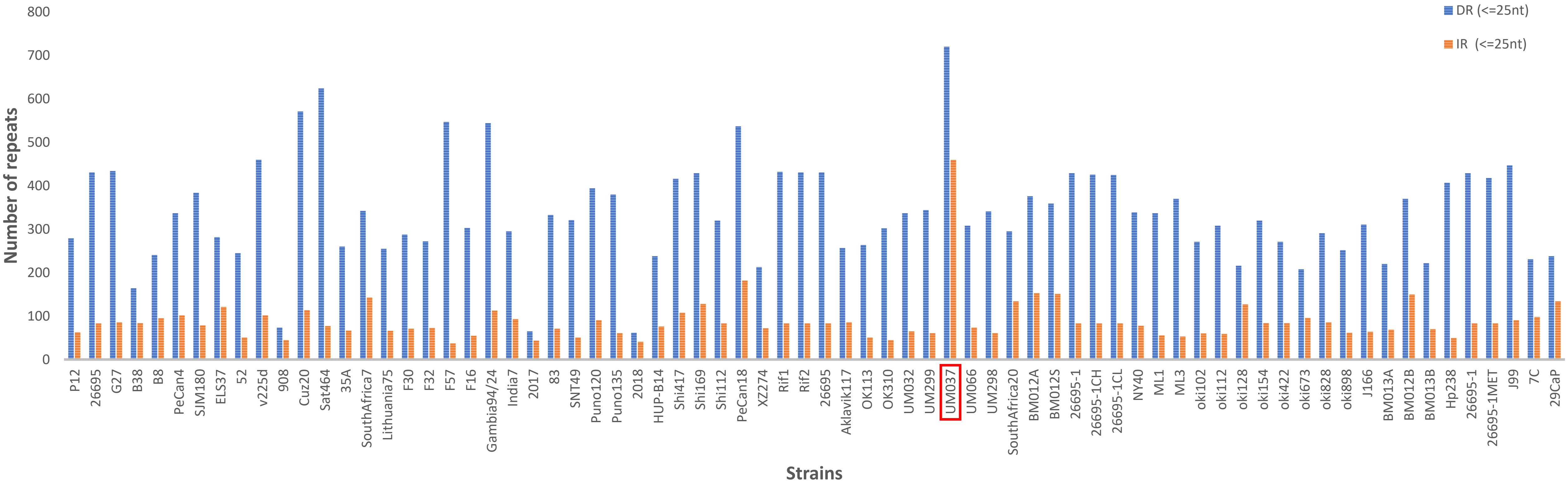

Figure S2. Occurrence of the direct and inverted repeats in each strain. Direct and inverted repeats of length >=25 nucleotide and 100 % sequence identity are shown. Strain UM037 had the highest number of direct as well as the inverted repeats among all of the analyzed strains.
