# Supplementary material for: Genetic Markers of Genome Rearrangements in Helicobacter pylori
Source: Microorganisms. 2021 Mar 17;9(3):621. doi: 10.3390/microorganisms9030621 (PMC8002640; doi:10.3390/microorganisms9030621)
Supplement: Supplementary file 1 [file microorganisms-09-00621-s001.zip › Supplementary_files/Supplementary file4_Figure S3.pdf]

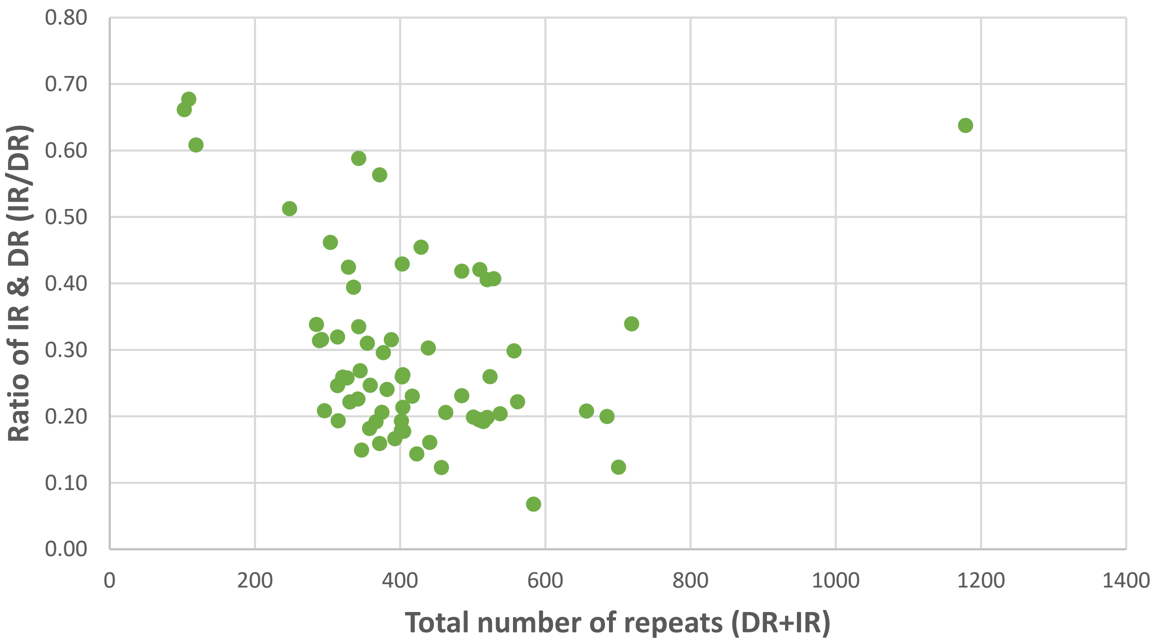

Figure S3: Distribution of the ratio of inverted repeats (IR) over direct repeats (DR). This ratio (IR/DR) less than 1 indicates the underrepresentation of inverted repeats.
