# Supplementary material for: Genetic Markers of Genome Rearrangements in Helicobacter pylori
Source: Microorganisms. 2021 Mar 17;9(3):621. doi: 10.3390/microorganisms9030621 (PMC8002640; doi:10.3390/microorganisms9030621)
Supplement: Supplementary file 1 [file microorganisms-09-00621-s001.zip › Supplementary_files/Supplementary file5_Figure S4.pdf]

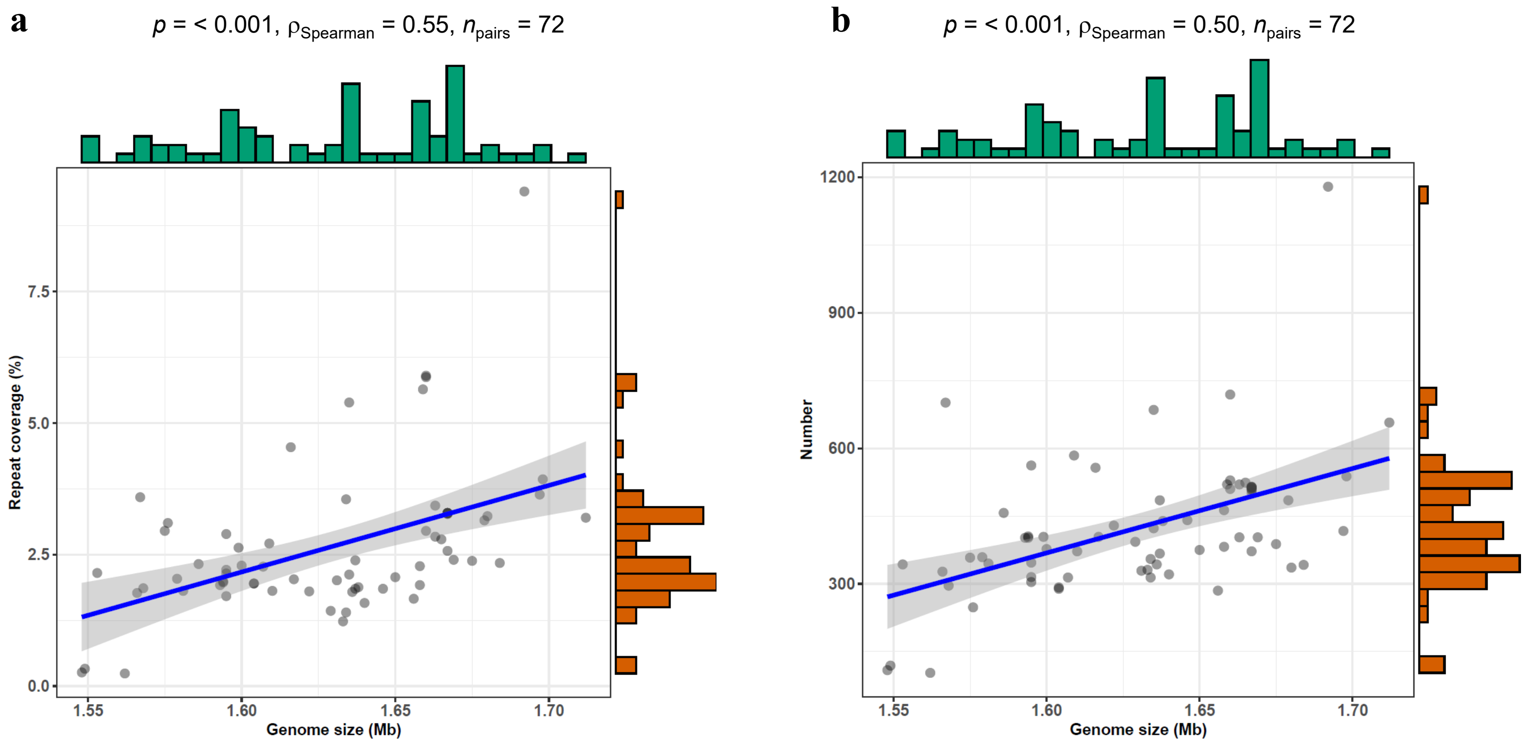

Figure S4. (a) Association between genome size and repeat coverage; **(b)** Association between genome size and number of repeats. Positive correlation was observed for both a and b.
