# Supplementary material for: Genetic Markers of Genome Rearrangements in Helicobacter pylori
Source: Microorganisms. 2021 Mar 17;9(3):621. doi: 10.3390/microorganisms9030621 (PMC8002640; doi:10.3390/microorganisms9030621)
Supplement: Supplementary file 1 [file microorganisms-09-00621-s001.zip › Supplementary_files/Supplementary file7_Figure S5.pdf]

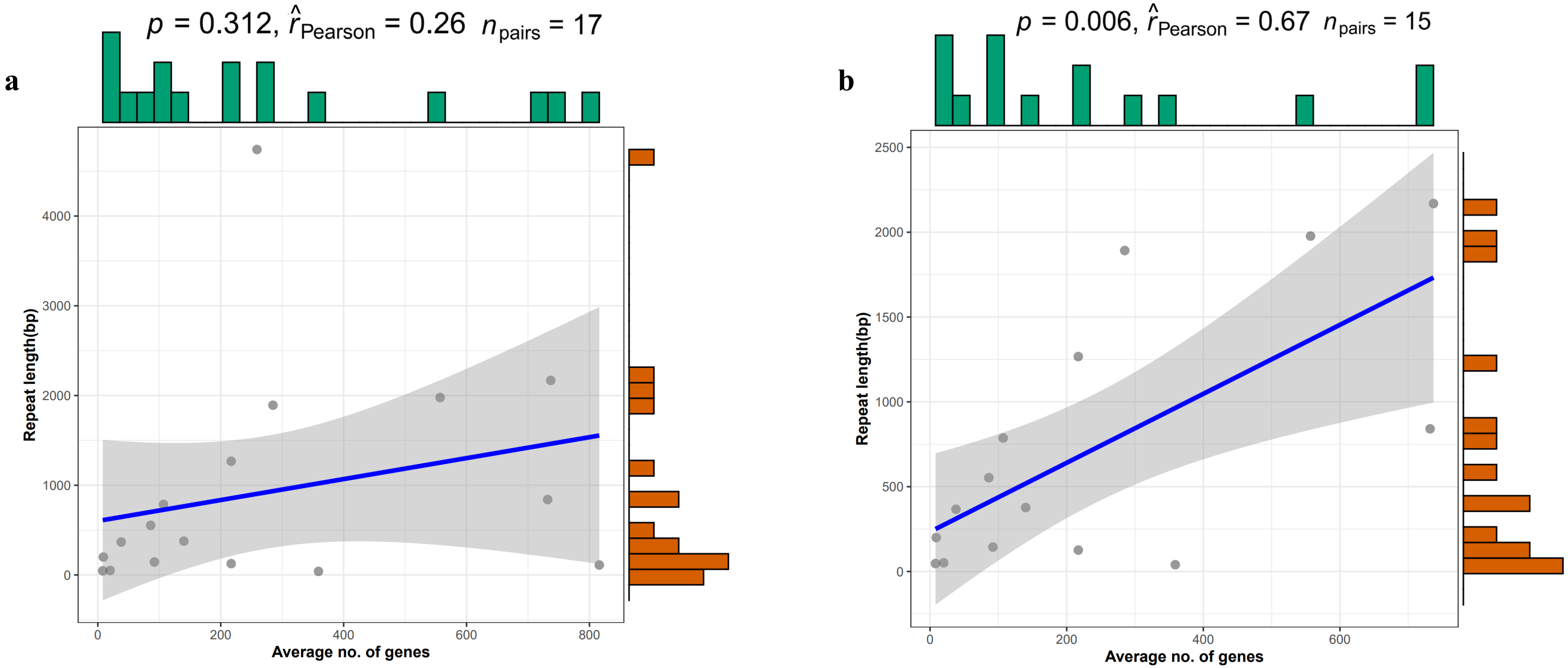

Figure S5. (a) Association between average number of genes in an inversion and length of repeat present around its breakpoints; (**b**) Association between average number of genes in an inversion and length of repeat present around its breakpoints after removing two outliers [the inversion R7 (the inverse transposition of 22 genes when dealt as an inversion had 816 genes while the repeat was 111 bp in length) and inversion R26 (strain-specific inversion)]. A significant positive correlation was observed.
