# Supplementary material for: Genetic Markers of Genome Rearrangements in Helicobacter pylori
Source: Microorganisms. 2021 Mar 17;9(3):621. doi: 10.3390/microorganisms9030621 (PMC8002640; doi:10.3390/microorganisms9030621)
Supplement: Supplementary file 1 [file microorganisms-09-00621-s001.zip › Supplementary_files/Supplementary file8_Figure S6.pdf]

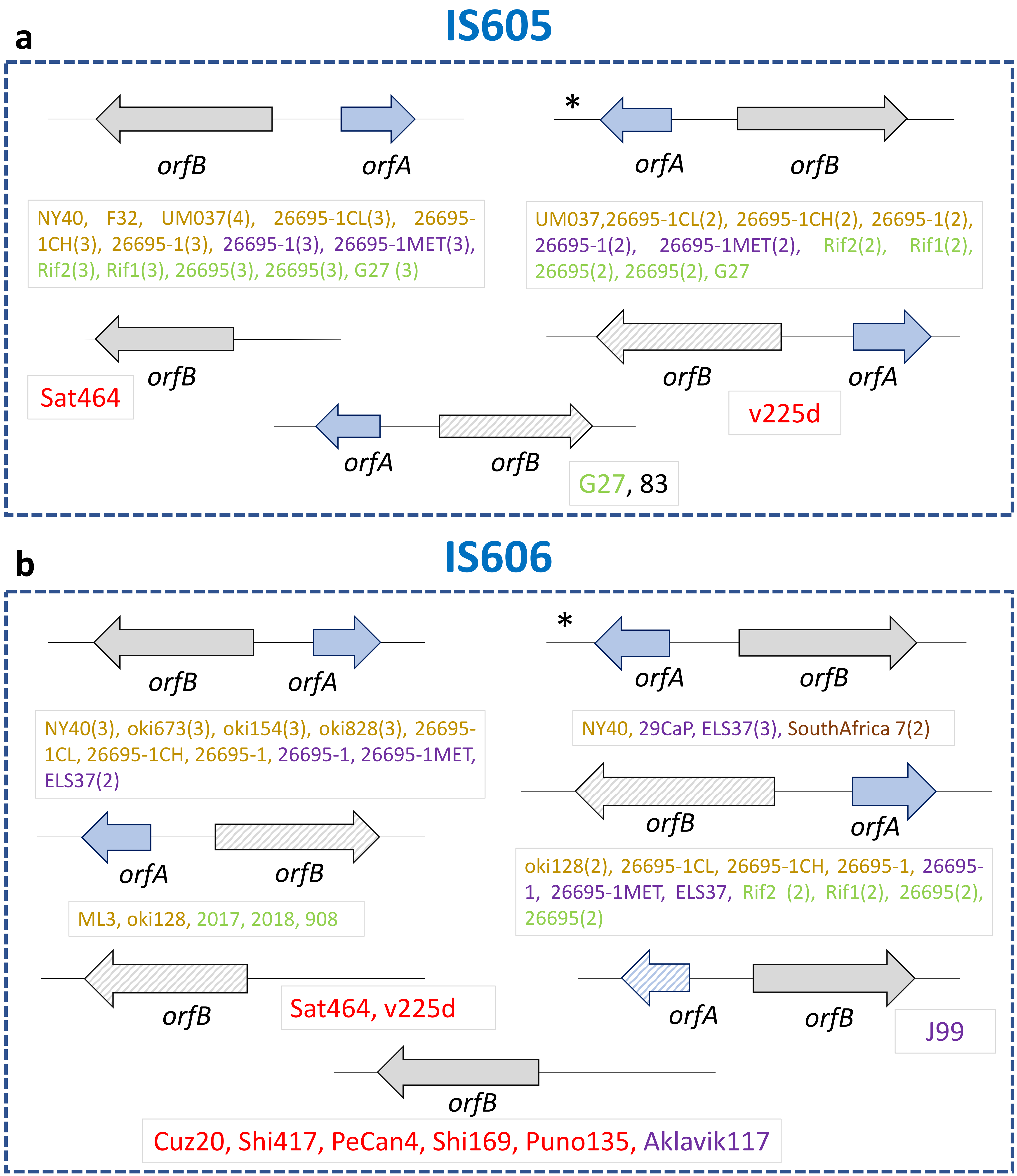
a
IS605
orfB
orfA
NY40, F32, UM037(4), 26695-1CL(3), 26695-1CH(3), 26695-1(3), 26695-1(3), 26695-1MET(3), Rif2(3), Rif1(3), 26695(3), 26695(3), G27 (3)
*
orfA
orfB
UM037,26695-1CL(2), 26695-1CH(2), 26695-1(2), 26695-1(2), 26695-1MET(2), Rif2(2), Rif1(2), 26695(2), 26695(2), G27
orfB
Sat464
orfB
orfA
v225d
orfA
orfB
G27, 83
b
IS606
orfB
orfA
NY40(3), oki673(3), oki154(3), oki828(3), 26695-1CL, 26695-1CH, 26695-1, 26695-1, 26695-1MET, ELS37(2)
*
orfA
orfB
NY40, 29CaP, ELS37(3), SouthAfrica 7(2)
orfA
orfB
ML3, oki128, 2017, 2018, 908
orfB
orfA
oki128(2), 26695-1CL, 26695-1CH, 26695-1, 26695-1, 26695-1MET, ELS37, Rif2 (2), Rif1(2), 26695(2), 26695(2)
orfB
Sat464, v225d
orfA
orfB
J99
orfB
Cuz20, Shi417, PeCan4, Shi169, Puno135, Aklavik117

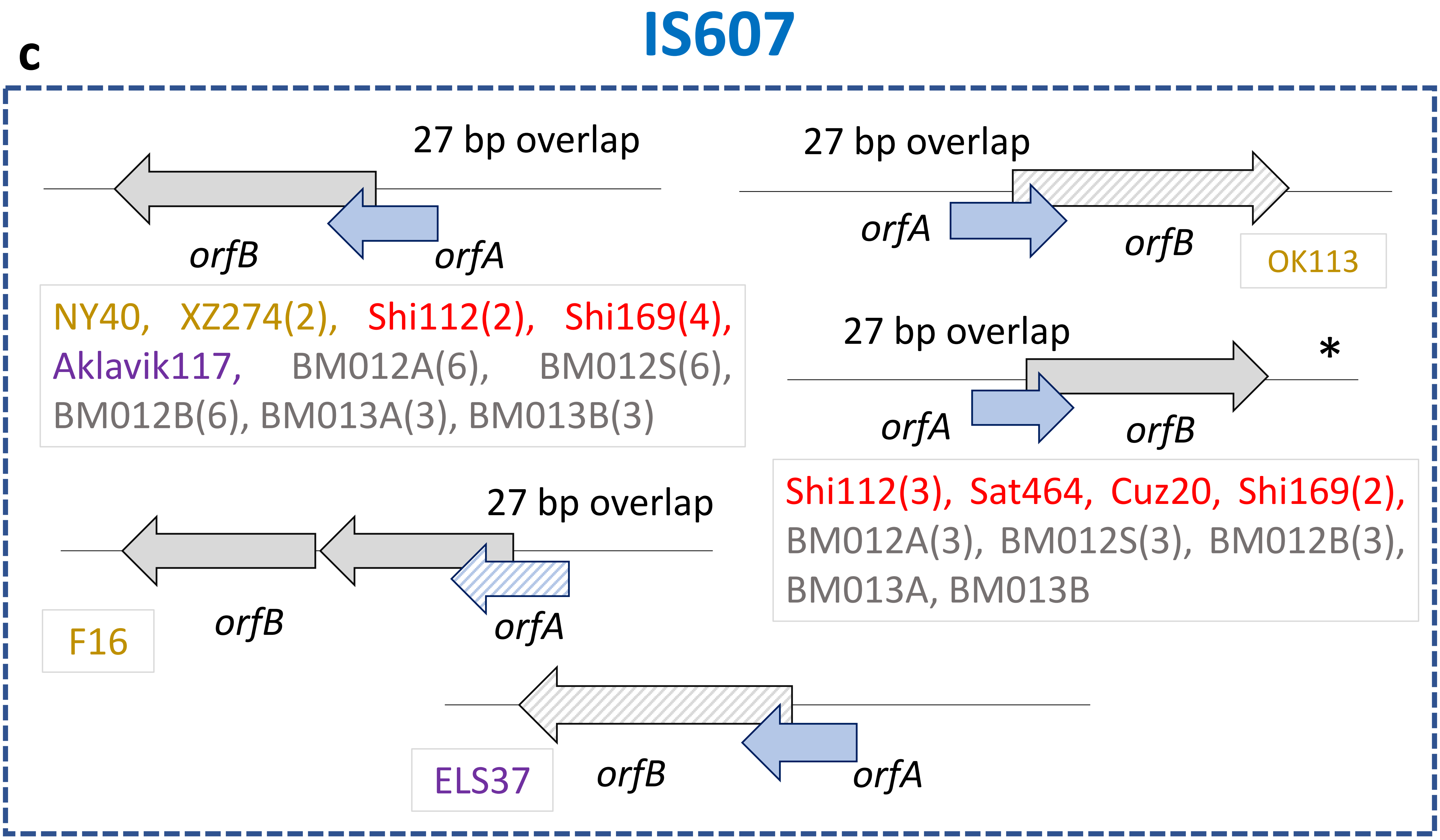
c
IS607
27 bp overlap
orfB
orfA
NY40, XZ274(2), Shi112(2), Shi169(4), Aklavik117, BM012A(6), BM012S(6), BM012B(6), BM013A(3), BM013B(3)
27 bp overlap
orfA
orfB
OK113
27 bp overlap
*
orfA
orfB
Shi112(3), Sat464, Cuz20, Shi169(2), BM012A(3), BM012S(3), BM012B(3), BM013A, BM013B
27 bp overlap
orfB
orfA
F16
orfB
orfA
ELS37

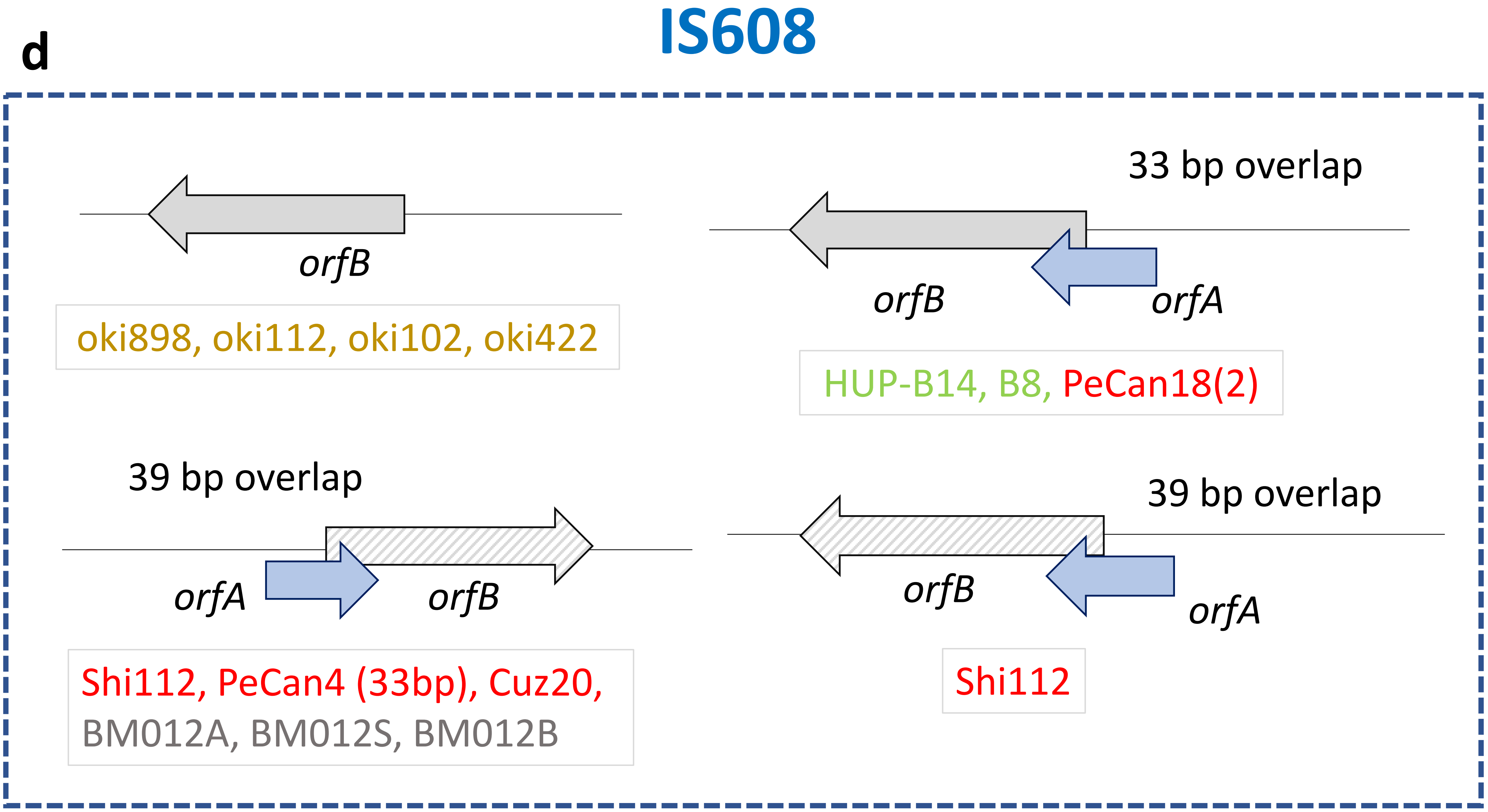
d
IS608
orfB
oki898, oki112, oki102, oki422
33 bp overlap
orfB
orfA
HUP-B14, B8, PeCan18(2)
39 bp overlap
orfA
orfB
Shi112, PeCan4 (33bp), Cuz20, BM012A, BM012S, BM012B
39 bp overlap
orfB
orfA
Shi112

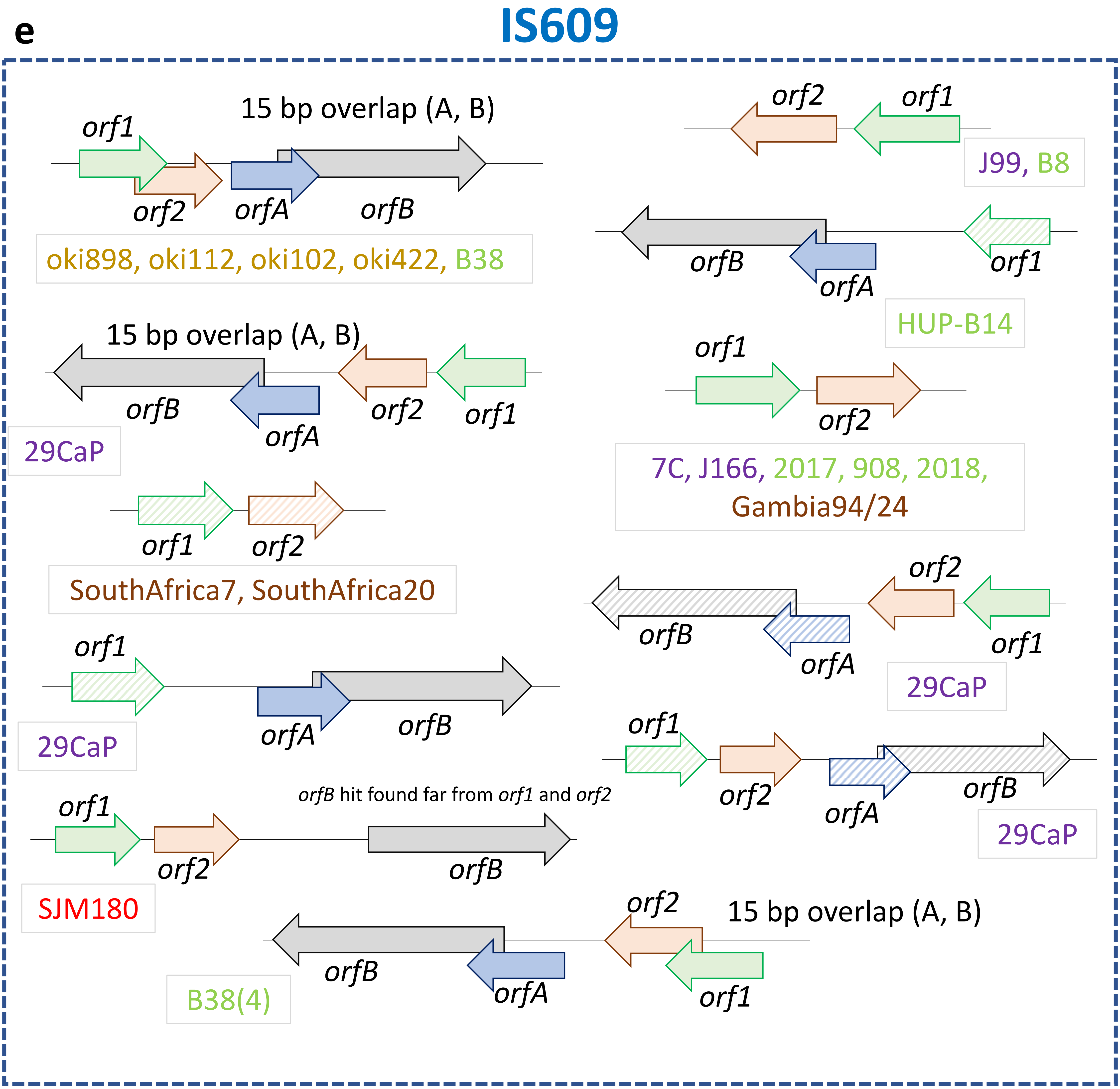

Figure S6. Structure of each insertion sequence found in strains from different geographical locations designated by different colors (East Asia, South America, North America, Australia, Europe, Africa). The number written next to each strain name represents the number of copies of IS element. The stripped arrows indicate that it is a pseud gene. Asterisk (*) represent the orientation as in ref [18, 19]. **(a)** IS605; **(b)** IS606; **(c)** IS607; **(d)** IS608; **(e)** IS609
